# Interaction of Laurusides 1 and 2 with the 3C-like Protease (M^pro^) from Wild-Type and Omicron Variant of SARS-CoV-2: A Molecular Dynamics Study

**DOI:** 10.3390/ijms24065511

**Published:** 2023-03-14

**Authors:** Ida Autiero, Giovanni N. Roviello

**Affiliations:** Institute of Biostructures and Bioimaging, Italian National Council for Research (IBB-CNR), Area di Ricerca Site and Headquarters, Via Pietro Castellino 111, 80131 Naples, Italy

**Keywords:** molecular dynamics, 3C-like protease, M^pro^, omicron, variants, SARS-CoV-2, COVID-19, bay laurel, phytochemistry, food drugs

## Abstract

*Laurus nobilis* (bay laurel) is a natural source of biological compounds, and some of its extracts and phytocompounds are also endowed with antiviral activity toward the family of the severe acute respiratory syndrome (SARS)-associated β-coronaviruses. Some glycosidic laurel compounds such as laurusides were proposed as inhibitors of important protein targets of SARS-CoV-2, which clearly recalls their potential as anti-COVID-19 drugs. Due to the frequent genomic variations of the β-coronaviruses and the consequent importance of evaluating a new drug candidate with respect to the variants of the target β-coronavirus, we decided to investigate at an atomistic level the molecular interactions of the potential laurel-derived drugs laurusides 1 and 2 (L01 and L02, respectively) toward a well-conserved and crucial target, the 3C-like protease (M^pro^), using the enzymes of both the wild-type of SARS-CoV-2 and of the more recent Omicron variant. Thus, we performed molecular dynamic (MD) simulations of laurusides—SARS-CoV-2 protease complexes to deepen the knowledge on the stability of the interaction and compare the effects of the targeting among the two genomic variants. We found that the Omicron mutation does not significantly impact the lauruside binding and that L02 connects more stably with respect to L01 in the complexes from both variants, even though both compounds prevalently interact within the same binding pocket. Although purely in silico, the current study highlights the potential role of bay laurel phytocompounds in the antiviral and specifically anti-coronavirus research and shows their potential binding toward M^pro^, corroborating the important commitment of bay laurel as functional food and disclosing novel scenarios of lauruside-based antiviral therapies.

## 1. Introduction

The two most serious pandemics of the 21st century were caused by β-coronaviruses: SARS in 2002–2003 was linked to SARS-CoV [1], while the 2019 coronavirus disease (COVID-19) [2] was caused by the severe acute respiratory syndrome coronavirus 2 (SARS-CoV-2), for which numerous prophylactic [3,4,5,6,7] and therapeutic [8,9,10,11,12,13,14] measures were investigated. COVID-19 has affected about 755 million people as of 7 February 2023 and more than 6.8 million people have died (according to https://covid19.who.int/, accessed on 7 February 2023). Despite the enormous efforts that the scientific research and the pharmaceutical companies have made in developing effective prophylactic and/or therapeutic agents, the efficiency of these measures is hampered by the frequent changes in the SARS-CoV-2 virus genome. Famously, vaccines trigger an immune response in the body, which is believed to be bolstered also by constant physical exercise [15], nature-inspired strategies [16,17,18,19], or following the consumption of vitamins and micronutrients [20]. However, SARS-CoV-2 has evolved to produce variant strains with varying levels of virulence and transmissibility including Alpha (B.1.1.7), Beta (B.1.351), Gamma (P.1), Delta (B.1.617.2), and Omicron (B.1.1.529) [21]. Notably, compared to other variants of SARS-CoV-2, Omicron is the most highly mutated, with 50 mutations across its genome, especially in the gene encoding for the spike (S) protein. These mutations help Omicron evade the immune barrier offered by the available immunizations [22]. Due to the high risk of infection and high reinfection rate, Omicron became the dominant variant worldwide. However, although it is highly contagious and spreads rapidly, epidemiological studies have shown that Omicron leads to less severe COVID-19 than wild-type strains and other variants of SARS-CoV-2 [23,24]. In any case, the likelihood of SARS-CoV-2 with its variants remaining around for an extended period of time, with serious consequences in elderly and frail patients and of new pathogenic β-coronaviruses appearing in the next decades, emphasizes the importance of developing safe and effective broad-spectrum anti-coronavirus drugs. In this context, the main protease (M^pro^) of SARS-CoV-2, also termed 3-chymotrypsin-like proteases (3CL^pro^), is an attractive therapeutic target due to the critical role it plays in viral replication and its conservation among β-coronaviruses and the different SARS-CoV-2 variants [25]. Thus, considering that the M^pro^ has a crucial role in the life cycle of all the human β-coronaviruses, this hydrolase is viewed as a promising target for developing broad-spectrum anti-coronavirus therapies [26] and, more specifically, accumulating evidence has consolidated an indispensable role for the M^pro^ targeting as a fundamental strategy for therapy of COVID-19. Importantly, the absence of any closely related M^pro^ homologs in humans makes M^pro^ inhibitors a first-choice class of COVID-19 drugs, which are unlikely to cause any serious side effects [26].

Structurally, the M^pro^ (34.21 kDa) monomer consists of three domains, including domain I, domain II, and domain III, with the latter one being an extra helix domain, whose aggregation initiates the M^pro^ dimerization in a homodimeric form, which is more active than the monomer and acts as the functional unit endowed with the highest hydrolytic function. Notably, the catalytic site is found at the intersection of domains I and II, which can be divided into five (S1–S5) sub-pockets, and only one of the two catalytic sites possesses hydrolytic activity in the homodimer. Different from 3-chymotrypsin whose activity is linked to a catalytic triad, the M^pro^ function is based on a catalytic dyad formed by His-41 and Cys-145 [26]. Apart from the catalytic dyad, the M^pro^ active site is demarcated by residues Ser-46, Gln-189, Thr-190, Ala-191, Pro-168, Glu-166, Leu-141, and Asn-142 [27].

To date, some synthetic drugs such as Nirmatrelvir, marketed as Paxlovid [28,29], as well as numerous natural molecules, have been identified as efficacious inhibitors of the M^pro^, displaying great potential for developing novel COVID-19 drugs and broad-spectrum anti-coronavirus therapeutics [26]. Among the several classes of compounds with importance as inhibitors of the M^pro^, those found in foods and especially plants of dietary use are given a great relevance. In fact, over the past few years, a number of structurally diverse plant molecules [30,31] and their derivatives have been found to have potential therapeutic effects [32], and some of them have been identified as antiviral molecules. In general, different studies have demonstrated the beneficial properties of culinary Mediterranean plants [33,34] and corresponding functional foods [35,36,37] with respect to several ailments. Some of them can strengthen the human body’s defenses against viral infections because they interfere with the life cycle of the virus or have beneficial effects on the host immunity [38,39,40,41], while others such as several steroidal glycosides including ouabain, digitoxin, and digoxin have shown in vitro a direct inhibitory activity against SARS-CoV-2 [42]. The influenza viruses and the coronavirus SARS-CoV-2 are only some examples of the many viral pathogens that can be managed using foods with antiviral properties [43] including bay laurel (*Laurus nobilis*), a culinary Mediterranean plant whose extracts were found to be active against highly pathogenic human coronaviruses such as SARS-CoV [44]. Remarkably, bay laurel contains specific glycosides known as laurusides [45] that were hypothesized to be inhibitors of SARS-CoV-2 M^pro^ [46] and, among these, laurusides 1 and 2 (herein below indicated as L01 and L02) showed high scores of the M^pro^ binding affinity [46], and enzyme inhibition as predicted by Molinspiration software (Table S1). Chemically, L01 and L02 are diastereomers showing comparable properties, as reported in Table S1.

**Scheme 1:**
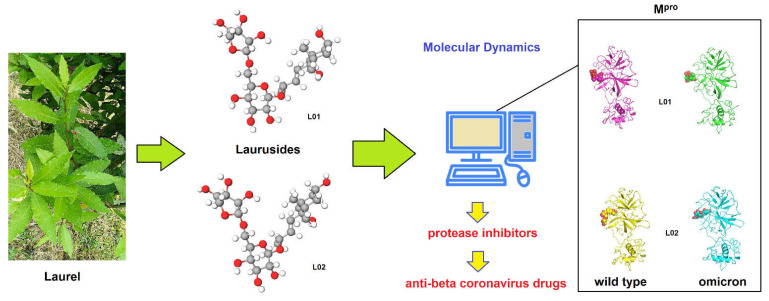
Schematic representation of the main methodology adopted in this work. Laurel-derived glycosides (laurusides) are investigated by molecular dynamics (MD) as potential binders of the M^pro^s of wild-type and Omicron SARS-CoV-2 in the context of the new anti-β-coronavirus drug discovery (photo taken by GN Roviello).

Pursuant to the above premise, we decided to investigate in the present work the interaction of laurusides L01 and L02 with the M^pro^ of both wild-type and Omicron variants of SARS-CoV-2 by using molecular dynamics simulations. Although purely in silico and lacking an experimental validation, this is a relatively rapid approach that makes it possible to obtain important information on new drug candidates (Scheme 1).

The main goals of this investigation were (i) to get more insights on the potential ability of these phytocompounds to interact with residues of the protease involved in its catalytic activity, potentially inhibiting a function that is of vital importance for virus replication; as well as (ii) to collect new elements supporting the hypothesis of a potential utility of bay laurel as a source of food drugs useful to prevent or mitigate the effects of the infection caused by SARS-CoV-2 and possibly other β-coronaviruses.

## 2. Results and Discussion

To investigate in silico the M^pro^ binding activities of laurusides L01 and L02, we modeled their respective interactions with the main proteases of wild-type and Omicron variants of SARS-CoV-2 (M^pro^WT and M^pro^O, respectively). Indeed, this molecular interaction could reinforce the hypothesis that laurusides are able to protect from the COVID-19 infection by inhibiting the M^pro^ activity already reported in [46] and could thus, support the use of bay laurel as functional food.

**Figure 1:**
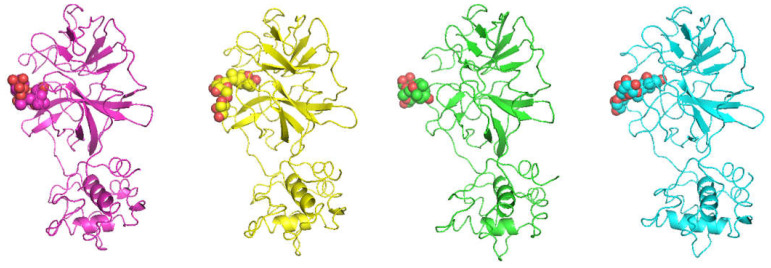
Wild-type and Omicron M^pro^ variants in complex with the L01 and L02 compounds. The protein structures are shown in cartoon and the compounds in spheres with the following scheme color: magenta: L01-M^pro^WT (W1), yellow: L02-M^pro^WT (W2), green: L01-M^pro^O (O1), cyan: L02─M^pro^O (O2).

The three-dimensional structure of L01 and L02 (depicted as 2D structures in Figure S1) were manually complexed with both the M^pro^ variants, using the Nirmatrelvir inhibitor [28,29] in complex with the M^pro^ of Omicron (carrying the P132H mutation) deposited in the 7TLL X-ray PDB structure [47] as guide to define the binding pose (Figure 1). In this complex structure, the ligand cyano moiety contacted Asn-142, Gly-143, Cys-145, and Glu-166 with its main binding residues seeming to be asparagine and cysteine. In detail, the models of the two M^pro^ variants were both superimposed to the 7TLL structure, and the binding pose of the Nirmatrelvir was used to define the binding pockets of the lauruside compounds.

**Figure 2:**
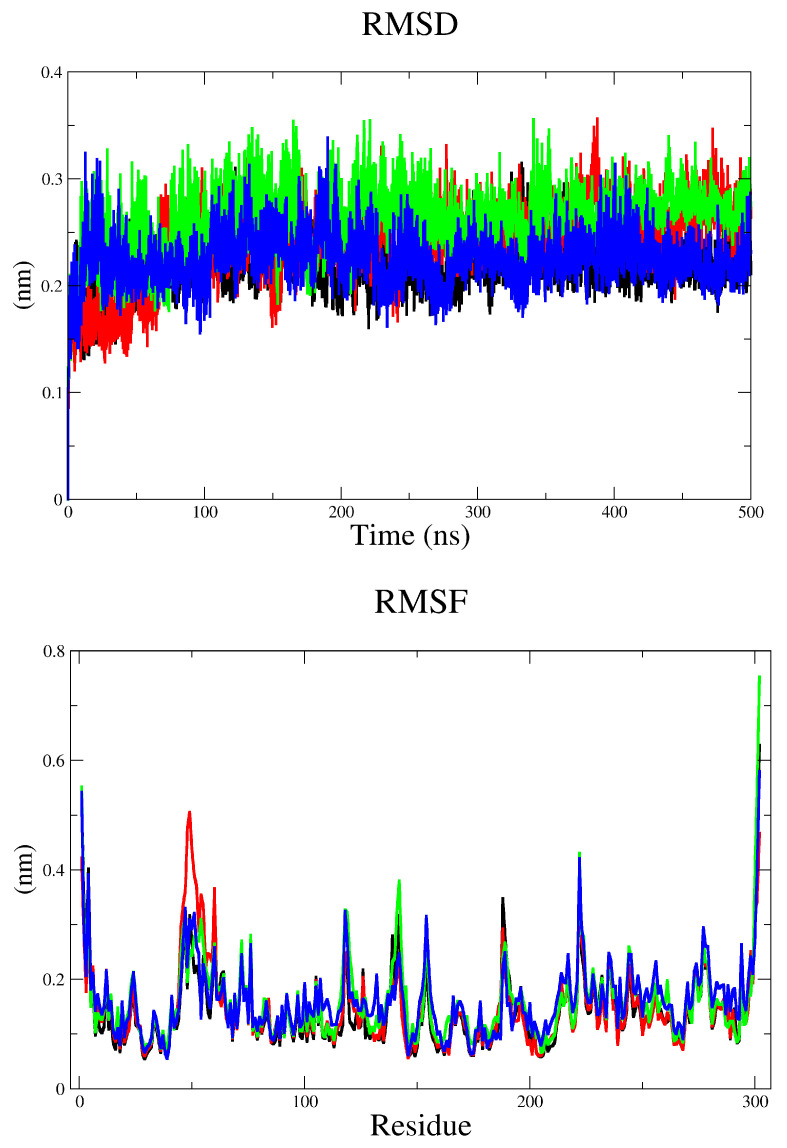
Top: C-α RMSD profiles along the L01─M^pro^WT, (mean: 0.22, SD: 0.02, black), L02─M^pro^WT (mean: 0.24 and SD 0.04, red), L01─M^pro^O (mean: 0.26 and SD: 0.03, green) and L02─M^pro^O (mean 0.22 and SD 0.02, blue) whole simulation complexes. Bottom: residue root-mean-square fluctuation (RMSF) profiles along the L01─M^pro^WT, (black), L02─M^pro^WT (red), L01─M^pro^O (green) and L02─M^pro^O (blue) whole simulation complexes.

The obtained four lauruside-M^pro^ complexes (hereafter W1, W2, O1, and O2 for L01 and L02 in complex with wild-type and Omicron SARS-CoV-2, respectively) were then subjected to 500 ns of MD simulation, to stabilize the models and to assess the efficacy of the two ligands in targeting the M^pro^s in that specific pocket. The root mean squared deviations (RMSD), computed considering the protein C-α atoms along the trajectories, were also measured to monitor the deviation of the proteins from the initial conformation (Figure 2).

**Figure 3:**
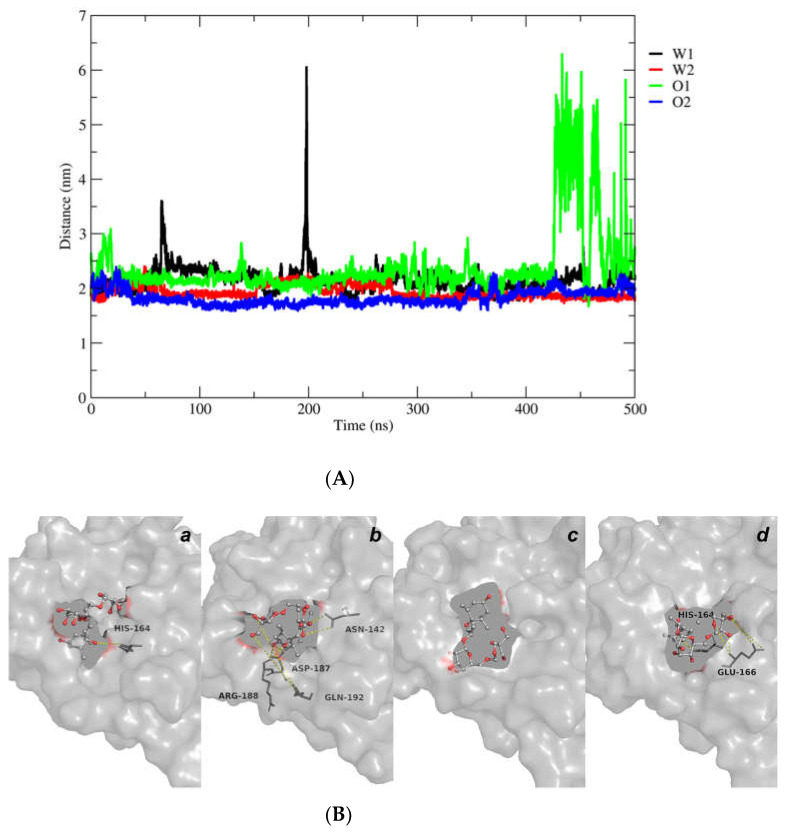
Protein-ligand center of masses distances along the L01-M^pro^WT, (black), L02-M^pro^WT (red), L01-M^pro^O (green) and L02-M^pro^O (blue) whole simulation complexes (**A**). Zoom view of the complex binding pose of the representative structures of M^pro^ variants in complex with L01 and L02. The protein structures are shown in transparent surface and the main protein residue interactors and the compounds in sticks and spheres. L01-M^pro^WT (**a**), L02-M^pro^WT (**b**), L01-M^pro^O (**c**) and L02-M^pro^O (**d**) (**B**).

This analysis suggests that the enzymes deviate in a comparable fashion along all the trajectories and the two ligands do not induce important M^pro^ conformation changes. Moreover, the RMSF plots of the protein residues were comparable along the four trajectories (Figure 2). Globally, the proteins are poorly affected by the presence either of L01 or L02 in both the variants. Although no significant overall changes of the three-dimensional structure of the protease were provoked by our ligands, their interaction with the M^pro^ could be still important as it may impede the virus infection by occupying an important active site of the protease structurally hindering the access to the natural substrates. Thus, we monitored the ligand-protein interactions by computing the persistence of the hydrogen bonds (Table 1 and Table 2) and the distances between the ligand and the protein center of masses along the four simulations (Figure 3A).

**Figure 4:**
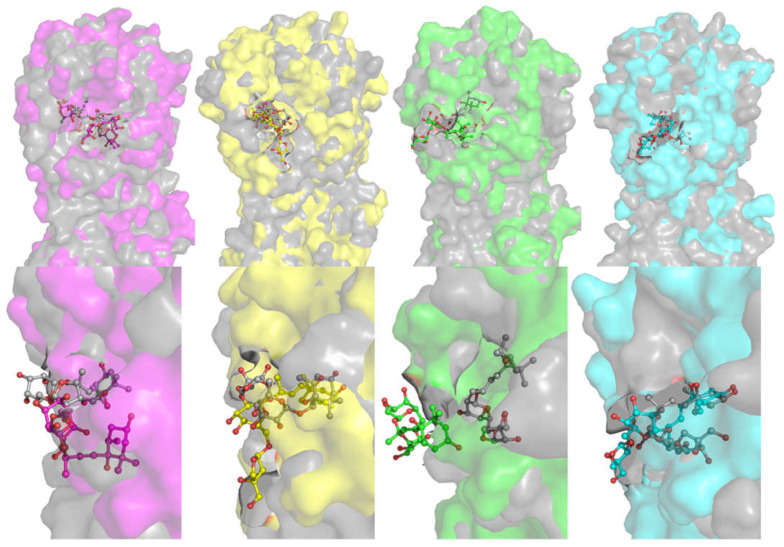
Superimposition of the starting and the representative structures of M^pro^ variants in complex with the L01 and L02 compounds. The proteins are shown in transparent surface and the compounds in sticks and spheres with the following scheme color: gray: starting trajectory structures, magenta: L01-M^pro^WT, yellow: L02-M^pro^WT, green: L01-M^pro^O, cyan: L02-M^pro^O.

In Table 1, the ligand-protein connections are reported as the percentage of hydrogen bonds occurrence among all the trajectory frames. L02 connects for more than 50% of the MD frames with the residues Asn-142, Arg-188, and Gln-192 in W2 and with Glu-166 in O2, but Asp-187 in W2 and His-164 in O2 are importantly connected to this ligand in more than 80% of the frames (Table 1). L01 shows a less conserved binding mode with more variable interaction networks of wider but poorly persistent connections and lower persistence of hydrogen bonds with either M^pro^WT than M^pro^O enzymes. In fact, this ligand detaches from the pocket during the last part of O1 simulation time (Figure 3A), while the other trajectories show the protein-ligand center-of-mass (COM, computing considering the overall protein and ligand atoms, respectively; for more details, please see Section 3) distance profiles to be comparable and rather stable. Even though this is somewhat expected given the nature of the targeting—indeed non-covalent interactions may induce loss-and-recapture events of the binding pose—such behavior is consistent with the lower stability of the L01-M^pro^ complexes relative to the L02 ones. In Figure 4, the representative complex conformations of each simulation, extracted from all the MD frames, are superimposed to the relative starting state to visualize the deviations from the initial pose of each system.

Consistent with the analysis described above, L02 well retains the initial position interacting with both M^pro^s. Conversely, L01 reveals a significant deviation from the initial binding region, particularly when it interacts with the Omicron M^pro^ protein variant. A detailed view of the most representative poses along the trajectories is also depicted in Figure 3B, where the main residues involved into the atomic interaction that persist for at least 20% of the trajectory frames along the MD are shown.

In summary, to explore at an atomistic level of details the M^pro^ binding ability of the laurusides, we here used MD simulations. During our calculations, one of the two ligands (L02) showed a more stable profile of interaction with both the M^pro^ variants with respect to the other ligand. In fact, L01 shows a less conserved targeting along the simulations, even if the binding is retained during most of the MD time. On the other hand, L02 can target the main SARS-CoV-2 protease stably accommodating in the same site occupied by Nirmatrelvir in its complex with the Omicron M^pro^ variant, as solved in the 7TLL PDB structure (Table 1) and used here as a template. Notably, L02 persists in the initial region for all simulations and, despite some rearrangements of the interaction network involving the sugar moieties, it stably connects to Asp-187 in wild-type and to Glu-166 in Omicron M^pro^ variants through persistent hydrogen bonds brought by the cyclohexane ring. In contrast, L01 exhibits a different targeting profile albeit differing only in the configuration of the secondary OH group on the cyclohexane moiety (Figure S1). To better clarify the different behavior of the two compounds, we evaluated the intramolecular hydrogen bonds within L01 and L02 (Table S2 and Figure S2). Notably, a higher number of persistent hydrogen bonds within the L01 with respect to L02, confirms that the last diastereomer is more prone to interacting with the protein, showing a not significant number of connections within the compound, except those among very close atoms. On the other hand, the diverse targeting properties shown by our ligands are represented somewhat already in the literature regarding diastereomers [48]. Overall, our investigations deepen important aspects of this molecular diversity, which often affects the efficacy of mixtures of compounds, often hiding the potential of the pure ligands as therapeutics. Natural compounds can be available in racemic or diastereoisomeric mixtures and the relative components’ efficacy needs to be carefully analyzed considering the diverse efficacy of the single components. We here show that a detailed picture of the interactions at the atomistic level is essential to suggest and guide novel applications of compounds extracted from natural products, such as lauruside derivatives, differing from each other for the configuration even of single stereocenters.

## 3. Methods

The three-dimensional structure of the wild-type and Omicron proteases were downloaded from the Protein Data Bank database (PDB: 6Y84 [49] and 7TLL [47], respectively). These structures were then used as templates to create whole models of the systems, filling the structural gaps, by homology modelling with MODELLER 9.22 program [50]. The three-dimensional structures of the two laurusides compounds were obtained by MOLVIEW (http://molview.org, accessed on 9 February 2023) in analogy to other literature examples [51,52], saved as .pdb files, and manually complexed into the M^pro^ variants using the binding pose of Nirmatrelvir in PDB 7TLL as a guide [47]. In particular, the coordinates of the Nirmatrelvir were used for the binding pocket definitions in order to insert both the laurusides, upon superimposition of the two variant models on the 7TLL X-ray structure of Nirmatrelvir in complex with Omicron M^pro^. The representative conformations were extracted from each MD simulation by the clustering method. MD simulations were run using the Parmbsc1 [53] with GROMACS 2020.6 [54] upon solvation of the systems in an octahedron box using the TIP3P [55] water models with a 1.1 nm distance to the border of the molecule, and 150 mM KCl concentration and additional ions for neutralization were added to simulate the biological conditions. The particle-mesh Ewald [56] method and Berendsen [57] algorithm were used to treat electrostatic interactions and control temperature and pressure, as already performed in previous protocols [58,59,60,61]. All the system waters were firstly relaxed by energy minimization and 10 ps of simulations at 300 K using protein and ligands atomic position restrains, with a harmonic potential. Successively, the systems gradually heated from 50 K to 300 K in a six step phases. Finally, simulation runs for 500 ns without restraints were preceded by NPT standard conditions for 2500 steps. The trajectories were analyzed using GROMACS [62] VMD [63] Grace [64] and Pymol [65] packages. Clustering analyses of each MD simulation were used to select representative conformations of each complex using the gromos clustering method with the algorithm presented by Daura et al., based on RMSD criteria [66]. The center-of-mass distances plotted in Figure 3A were computed by using GROMACS tools; we also reported as comparison the distance between the Omicron M^pro^ protein and Nirmatrelvir center of masses of the X-ray structure 7TLL (Figure S3). Note that Nirmatrelvir belongs to the class of the synthetic peptide derivatives [67,68]. The enzymatic score for each lauruside was computed by Molinspiration (Molinspiration Cheminformatics free web services, Slovensky Grob, Slovakia; https://www.molinspiration.com/ accessed on 7 February 2023) using the following Simplified Molecular Input Line Entry System (SMILES) codes:

L01 [IUPAC name: *(2R,3E)-4-[(1S,4R,6R)-1,4-Dihydroxy-2,2,6-trimethylcyclohexyl]-3-buten-2-yl 6-O-[(2R,3R,4R)-3,4-dihydroxy-4-(hydroxymethyl)tetrahydro-2-furanyl]-β-D-glucopyranoside* ] SMILES:
C[C@H](C=C[C@@]1(O)[C@H](C)C[C@@H](O)CC1(C)C)O[C@@H]3O[C@H](CO[C@@H]2OC[C@](O)(CO)[C@H]2O)[C@@H](O)[C@H](O)[C@H]3O
L02 [IUPAC name: *(2R,3E)-4-[(1S,4S,6R)-1,4-Dihydroxy-2,2,6-trimethylcyclohexyl]-3-buten-2-yl 6-O-[(2R,3R,4R)-3,4-dihydroxy-4-(hydroxymethyl)tetrahydro-2-furanyl]-β-D-glucopyranoside* ] SMILES:
C[C@@H]1C[C@@H](CC([C@]1(/C=C/[C@@H](C)O[C@H]2[C@@H]([C@H]([C@@H]([C@H](O2)CO[C@H]3[C@@H]([C@](CO3)(CO)O)O)O)O)O)O)(C)C)O

More details on the enzymatic inhibition score and other useful information achievable through Molinspiration are given at https://www.molinspiration.com/docu/miscreen/druglikeness.html (accessed on 7 February 2023).

## 4. Conclusions

Aiming to provide more efficacious COVID-19 drugs useful also as broad-spectrum anti-coronavirus agents [69], in this work we focused on the structural features of the binding of certain almost unexplored phytomolecules, i.e., the laurusides extracted from bay laurel, toward one of the main therapeutic targets investigated in the fight against pathogenic human β-coronaviruses, i.e., the main protease M^pro^. More in detail, we examined the binding behavior of two glycosidic compounds found in bay laurel, herein referred to as L01 and L02, toward the M^pro^ of both wild-type and Omicron SARS-CoV-2. Molecular dynamics studies showed that M^pro^-L01 complexes show more variable interaction networks of wider but poorly persistent connections and lower persistence of hydrogen bonds with either the protease of the wild-type virus or the protease of the Omicron mutation. On the other hand, L02 is able to target the main SARS-CoV-2 protease stably accommodating in the same site occupied by Nirmatrelvir in its complex with the Omicron M^pro^ variant. Overall, our ligands, and especially L02, connect to the protein region deputed to accommodate the physiological substrates of the protein; indeed, specific residues of the M^pro^ catalytic site, such as Asn-142, and Glu-166, are also persistently interacting with laurusides. In summary, our analyses shed light on crucial structural points of targeting essential in the interaction of the laurel glycosides with the main protease of both the wild-type and Omicron variant of SARS-CoV-2. From a more applicative perspective, this study highlights not only the importance of laurusides from *Laurus nobilis* as M^pro^ ligands able to potentially modulate the protease activity with possible anti-COVID therapeutic effects, but it also supports the current importance given to bay laurel as a functional food that is part of the Mediterranean diet.

## Figures and Tables

**Table 1:** Percentage of occurrence of ligand-protein residue connections along the trajectory frames. For each pair, the sum of the hydrogen bonds between any residue atoms is considered.

Occurrence (%)					
		W1	W2	O1	O2
Thr	24	**8**			
Thr	25			**8**	
Thr	26	**12**			
His	41*	**8**		**5**	**9**
Thr	45			**3**	
Ser	46	**11**		**3**	
Tyr	54	**17**			
Met	49			**16**	
Asn	142	**13**	**58**		**13**
Gly	143	**2**			**13**
Cys	145 *				**4**
His	164 *	**20**		**7**	**68**
Glu	166 *	**24**	**18**	**2**	**83**
Asp	187		**100**		
Arg	188		**63**	**2**	**13**
Gln	189*	**23**	**5**	**41**	**2**
Thr	190			**12**	**7**
Gln	192		***73***	**11**	**9**

* appendix indicates residues taking part to the Nirmatrelvir binding site in the 7TLL PDB structure.

**Table 2:** Percentage of occurrence of ligand-protein connections along the trajectory frames, for each atomic pair. Only the connections present in more than 20% of the frames are reported.

MD	Protein Atom			Ligand Atom		Occurrence (%)
**w1**	His	164 *	O	L01	O2/O3	**20**
**w2**	Asn	142	ND2	L02	O1	**26**
	Asn	142	OD1	L02	O4/O57/O8	**32**
	Asp	187	OD1/2	L02	O2	**100**
	Arg	188	O	L02	O3	**63**
	Gln	192	O	L02	O9	**32**
	Gln	192	N	L02	O8	**34**
**o1**	-	-	-	-	-	**-**
**o2**	His	164 *	ND1	L02	O2	**66**
	Glu	166 *	OE1/2	L02	O8/O9	**21**
	Glu	166 *	N	L02	O10	**33**
	Glu	166 *	N	L02	O7	**21**

* appendix indicates residues taking part to the Nirmatrelvir binding site in the 7TLL pdb structure.

## Data Availability

Not applicable.

## Supplementary Materials

The following supporting information can be downloaded at: https://www.mdpi.com/article/10.3390/ijms24065511/s1.
