# Supplementary material for: Interaction of Laurusides 1 and 2 with the 3C-like Protease (Mpro) from Wild-Type and Omicron Variant of SARS-CoV-2: A Molecular Dynamics Study
Source: Int J Mol Sci. 2023 Mar 14;24(6):5511. doi: 10.3390/ijms24065511 (PMC10054487; doi:10.3390/ijms24065511)
Supplement: Supplementary file 1 [file ijms-24-05511-s001.zip › ijms-2267219-supplementary.pdf]

Supporting Information

Interaction of Laurusides 1 and 2 with the 3C-like Protease (Mpro) from Wild-Type and Omicron Variant of SARS-CoV-2: A Molecular Dynamics Study

Ida Autiero * and Giovanni N. Roviello *

Institute of Biostructures and Bioimaging, Italian National Council for Research (IBB-CNR), Area di Ricerca Site and Headquarters, Via Pietro Castellino 111, 80131 Naples, Italy
* Correspondence: ida.autiero@cnr.it (I.A.); giroviel@unina.it (G.N.R.)

Table S1. Structural representation and predicted properties of Laurusides L01 and L02

compound	Structure*	PAINS**	Enzyme Inhibitor score***	ClogP***	Number of violations of Lipinski's rule of five
Lauruside 1 (L01)	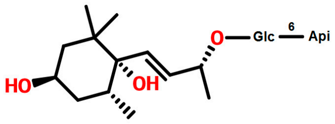	N	0.73	-1.06	3
Lauruside 2 (L02)	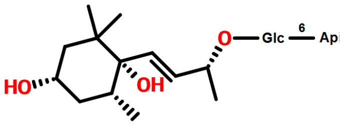	N	0.73	-1.05	3

*Glc: D-Glucopyranose; Api: 3-C-(Hydroxymethyl)-D-erythofuranose (Apiose) **Pan-assay interference compounds (PAINS) score and consensus logP (ClogP) were calculated with SwissADME[48]. ***Computed by Molinspiration

Table S2. Percentage of occurrence of intra-ligand interactions along the trajectory frames.

MD	atom pair	occurrence (%)
**w1**	O4-O5	69
	O8-O	62
**w2**	O3-O8	30
**o1**	O4-O5	71
	O8-O	65
**o2**	O4-O6	76

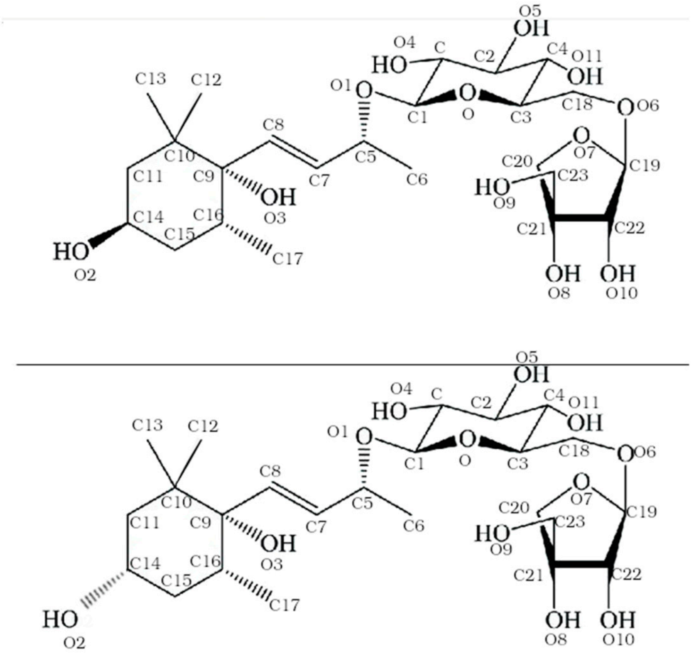

Figure S1. 2D Structural representation of L01 (top) and L02 (bottom) compounds with labelled atom numbering.

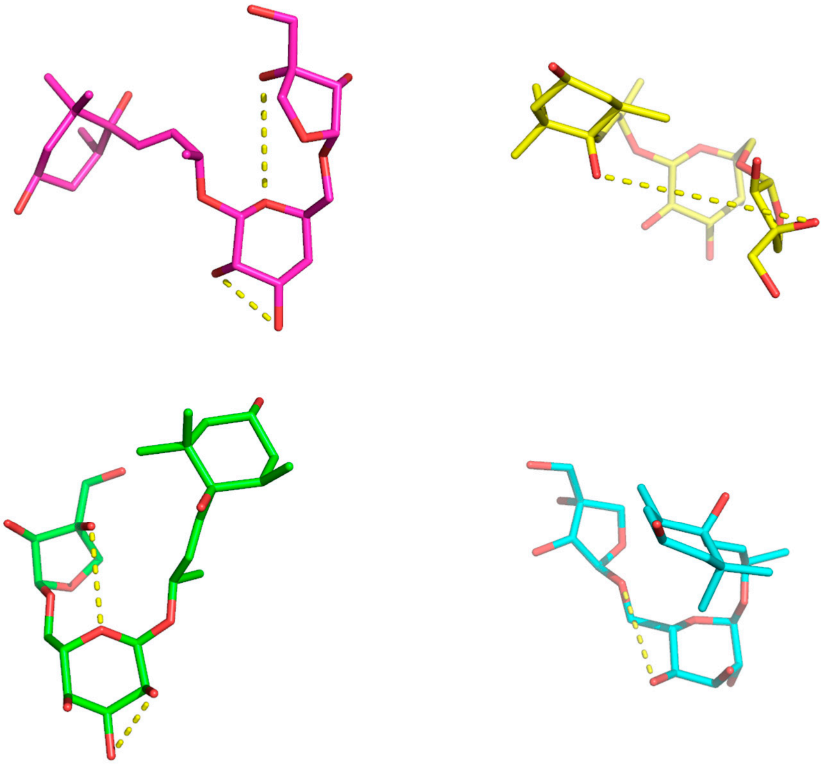

Figure S2. Representative structures of L01 and L02 compounds extracted from the four MD simulations. The compounds are shown in sticks with the following scheme color or carbon atoms: magenta: L01—M^{pro}WT, yellow: L02—M^{pro}WT, green: L01—M^{pro}O, cyan: L02—M^{pro}O.

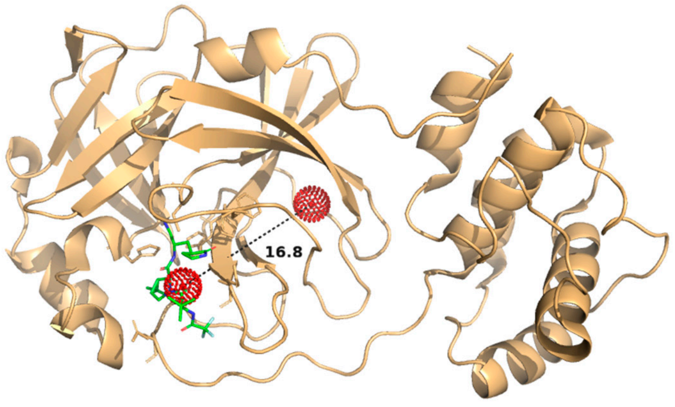

Figure S3. Distance between the overall atom omicron M^{pro} protein and Nirmatrelvir center of masses in the 7TLL X-ray structure. The protein is shown in sand cartoon and the Nirmatrelvir in red sticks colored according to the atoms (green: carbons, red: oxygens and blue: nitrogens) the center of masses are shown in red dots. The distance is expressed in nm.
